# Altered Goblet Cell Differentiation and Surface Mucus Properties in Hirschsprung Disease

**DOI:** 10.1371/journal.pone.0099944

**Published:** 2014-06-19

**Authors:** Jay R. Thiagarajah, Hasan Yildiz, Taylor Carlson, Alyssa R. Thomas, Casey Steiger, Alberto Pieretti, Lawrence R. Zukerberg, Rebecca L. Carrier, Allan M. Goldstein

**Affiliations:** 1 Department of Gastroenterology and Nutrition, Boston Children’s Hospital, Boston, Massachusetts, United States of America; 2 Department of Chemical Engineering, Northeastern University, Boston, Massachusetts, United States of America; 3 Department of Pediatric Surgery, Massachusetts General Hospital, Boston, Massachusetts, United States of America; 4 Department of Pathology, Massachusetts General Hospital, Boston, Massachusetts, United States of America; University of Southern California, United States of America

## Abstract

Hirschsprung disease-associated enterocolitis (HAEC) leads to significant mortality and morbidity, but its pathogenesis remains unknown. Changes in the colonic epithelium related to goblet cells and the luminal mucus layer have been postulated to play a key role. Here we show that the colonic epithelium of both aganglionic and ganglionic segments are altered in patients and in mice with Hirschsprung disease (HSCR). Structurally, goblet cells were altered with increased goblet cell number and reduced intracellular mucins in the distal colon of biopsies from patients with HSCR. Endothelin receptor B (Ednrb) mutant mice showed increased goblet cell number and size and increased cell proliferation compared to wild-type mice in aganglionic segments, and reduced goblet cell size and number in ganglionic segments. Functionally, compared to littermates, Ednrb^−/−^ mice showed increased transepithelial resistance, reduced stool water content and similar chloride secretion in the distal colon. Transcript levels of goblet cell differentiation factors SPDEF and Math1 were increased in the distal colon of Ednrb^−/−^ mice. Both distal colon from Ednrb mice and biopsies from HSCR patients showed reduced Muc4 expression as compared to controls, but similar expression of Muc2. Particle tracking studies showed that mucus from Ednrb^−/−^ mice provided a more significant barrier to diffusion of 200 nm nanoparticles as compared to wild-type mice. These results suggest that aganglionosis is associated with increased goblet cell proliferation and differentiation and subsequent altered surface mucus properties, prior to the development of inflammation in the distal colon epithelium. Restoration of normal goblet cell function and mucus layer properties in the colonic epithelium may represent a therapeutic strategy for prevention of HAEC.

## Introduction

Hirschsprung disease (HSCR) is a congenital disorder of the intestinal tract that is characterized by variable lengths of colorectal aganglionosis resulting from the failure of neural crest-derived cells to form the distal enteric nervous system (ENS) [1], [2]. Although the most obvious consequence resulting from the absence of enteric ganglia is a defect in colonic motility, the most serious complication is the development of Hirschsprung-associated enterocolitis (HAEC). HAEC is an inflammatory colitis that causes distension, diarrhea, and fever and can lead to bacterial translocation, sepsis, and death. The pathogenesis of HAEC remains unknown although several theories have been proposed, including changes to epithelial barrier properties, mucus layer properties, innate immunity and colonic microbiota composition [3], [4], [5]. In patients with HSCR, enterocolitis occurs despite surgery to remove the aganglionic bowel, suggesting that the pathogenic mechanisms involved in HAEC extend beyond the region of aganglionosis.

A luminal mucus layer coats the surface of the epithelium throughout the colon and is thought to be an important barrier against bacterial and viral infection and an important factor for maintaining the integrity of the epithelium [6]. The mucus layer consists of a mixture of water, complex glycosylated molecules (mucins), and antimicrobial proteins [7]. Studies of the mucus layer in HSCR have shown changes in both mucins and secreted immunoglobulin in patients with HAEC [8], [9], [10]. A study of mucin turnover in HSCR patients showed that development of enterocolitis was specifically related to an increase in the ratio of intracellular to secreted mucins [11]. The major mucus proteins are produced in goblet cells within the gut epithelium [12]. Goblet cells derive from a common secretory cell progenitor through suppression of the Notch pathway via Hes1 and expression of Math1 (murine homolog of Hath1 and also known as Atoh1) [13], [14], [15]. Terminal differentiation requires expression of SPDEF, an ETS family transcription factor that regulates goblet cell gene expression and is upregulated in goblet cell hyperplasia in the colon, prostate, and lung [16], [17], [18], [19], [20]. Goblet cell differentiation factors are altered in chronic inflammatory bowel diseases although different patterns of expression have been reported in Crohn’s disease versus ulcerative colitis. Increases in goblet cell number have been found in quiescent Crohn’s disease epithelium in contrast to a number of studies that show decreased goblet cell numbers in active Crohn’s disease and in ulcerative colitis [21], [22], [23]. The goal of this study was to determine how goblet cell and mucus layer structure and function are altered in aganglionic colon from mice and humans.

Endothelin receptor B (Ednrb) mutant mice are a well-described model of colorectal aganglionosis that exhibit many of the features of human HSCR including megacolon and development of HAEC [24], [25]. Ednrb^−/−^ mice die at age 3–5 weeks from either distal intestinal obstruction or HAEC-associated sepsis [25]. HAEC onset occurs typically soon after weaning (P21), with rapid progression thereafter. We previously showed that Ednrb^−/−^ mice exhibit early alterations in the normal colonic and fecal microbiome prior to the onset of significant colitis [26]. In this study we looked at the colonic epithelium from Ednrb^−/−^ mice and from biopsies obtained from HSCR patients without active inflammation, to investigate epithelial changes associated with aganglionosis. Our findings reveal early alterations in goblet cell differentiation and proliferation in aganglionic colon in HD patients and Ednrb^−/−^ mice. We also show that colonic barrier function, fluid absorption and secretion is initially unimpaired in the aganglionic colon. We therefore suggest that the production of a functionally altered mucus layer in the setting of colonic aganglionosis, likely in conjunction with alterations in the colonic microbiome, may play a key role in the pathogenesis of HAEC.

## Results

### Crypt Proliferation is Increased in Ednrb^−/−^ Mice

**Figure 1:**
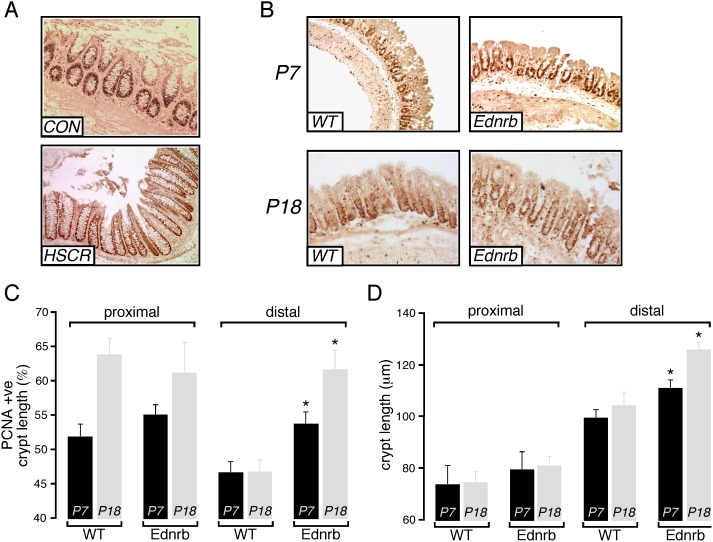
Colon crypt proliferation in Ednrb^−/−^ mice. A. Representative images showing PCNA staining of crypt cells from human distal colon from control (CON) and HSCR patients. B. Representative images showing PCNA staining of crypt cells from distal colon of P7 and P18 WT and Ednrb^−/−^ mice. C. Summary graph showing extent of PCNA-immunoreactive zone (% of total crypt length that is PCNA-positive) in the epithelium of proximal and distal colon from WT (n = 5) and Ednrb^−/−^ mice (n = 5). D. Mean crypt length in proximal and distal colon from WT (n = 5) and Ednrb^−/−^ mice (n = 5). *p<0.05.

Colon tissue was obtained from infants with a diagnosis of HSCR and from similar age controls with constipation and normal ganglion cells on biopsy (Table 1). The proximal extent of aganglionosis varied in the HSCR patients from recto-sigmoid to total colon with mucosal tissue obtained from aganglionic segments. Histological grading of inflammation in the biopsies showed no inflammation in HSCR colon, and none had any clinical evidence of HAEC. Initial studies of the human tissue suggested an expanded PCNA-positive proliferative zone in the aganglionic segment (Fig. 1A), but variability between samples limited meaningful quantitative comparison. We therefore investigated whether the finding was consistently present in Ednrb^−/−^ mice. In agreement with our initial observations in human HSCR, the distal colon of Ednrb^−/−^ mice showed increased thickness of the mucosal layer compared to WT littermates. Analysis of crypt proliferation in Ednrb^−/−^ mice was performed by PCNA staining of tissue samples. We found a significant expansion of the proliferative zone in the distal colon of Ednrb^−/−^ mice at both P7 and P18 as compared to WT littermates (Fig. 1B, C). Total crypt length was also significantly increased distally in Ednrb^−/−^ compared to WT colon both at P7 (111 µm vs 99 µm, p<0.05) and P18 (126 µm vs 104 µm, p<0.05) (Fig. 1D). In contrast, crypt length and proliferation in the proximal colon were not significantly different between mutant and WT. (Fig. 1C, D).

**Table 1:** Clinical parameters of human HSCR samples.

	Control	HSCR
Number	4	13
Mean age (weeks)	8	12
Extent of aganglionosis	N/A	Rectosigmoid: 5 Descending colon: 4 Total colon: 4

### Colonic Goblet Cell Numbers are Increased in Patients with HSCR and in the Distal Colon of Ednrb^−/−^ Mice

**Figure 2:**
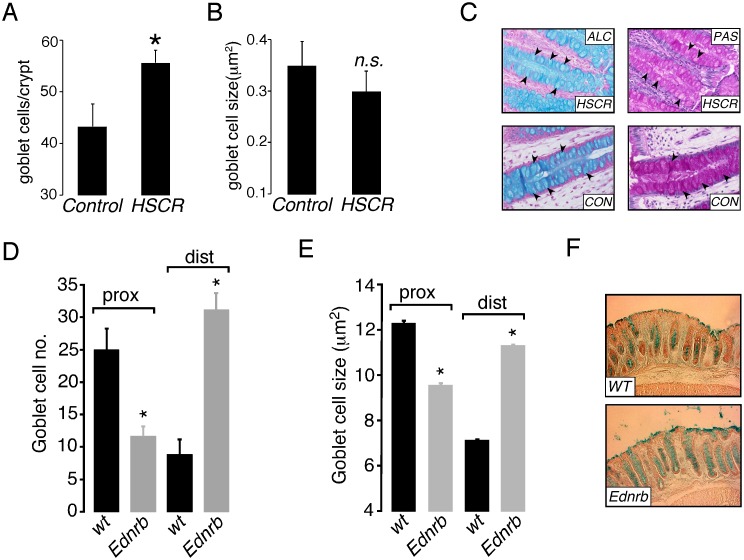
Colon goblet cells in human HSCR and Ednrb^−/−^ mice. A. Summary graph showing goblet cell density in colonic crypts from control (n = 4) and HSCR patients (n = 13). B. Summary graph showing goblet cell size (µm^2^) in colonic crypts from control (n = 4) and HSCR patients (n = 13). C. Representative images showing Periodic Acid Schiff (PAS) and Alcian blue (ALC) staining of colonic crypts from biopsies obtained from control (CON) and HSCR patients. D. Summary graph showing goblet cell density in colonic crypts from proximal (prox) and distal (dist) colon from WT (n = 5) and Ednrb^−/−^ mice (n = 5). E. Summary graph showing goblet cell size (µm^2^) in colonic crypts from WT (n = 5) and Ednrb^−/−^ mice (n = 5). F. Representative images showing Alcian blue staining of goblet cells from distal colon from WT and Ednrb^−/−^ mice. *p<0.05, n.s. = not significant.

Analysis of histology from human HSCR colon showed that goblet cell numbers were increased compared to controls (Figure 2A), while overall goblet cell size was similar (Fig. 2B). Qualitatively, both PAS and Alcian blue staining were less marked (Fig. 2C) in HSCR tissue, suggesting decreased amounts of both neutral and acidic goblet cell mucins.

In order to investigate the colonic epithelium more closely, distal and proximal sections of colon were analyzed from Ednrb^−/−^ mice at P18, prior to the development of enterocolitis. As shown in Fig. 2D–F, both goblet cell number and size were significantly increased in the distal (aganglionic) colon in Ednrb^−/−^ mice. Interestingly, an opposite effect was seen in the ganglionic proximal colon, where a reduction in goblet cell number and size was observed. Histological grading of inflammation and expression of the inflammatory cytokine IL-6, measured by qPCR, was not different between WT and Ednrb^−/−^ mice (data not shown), confirming that the Ednrb^−/−^ colon at this early time point did not exhibit inflammation.

### Transepithelial Resistance and Fecal Dehydration are Increased and Chloride Secretion is Similar in the Distal Colon of Ednrb^−/−^ Mice

**Figure 3:**
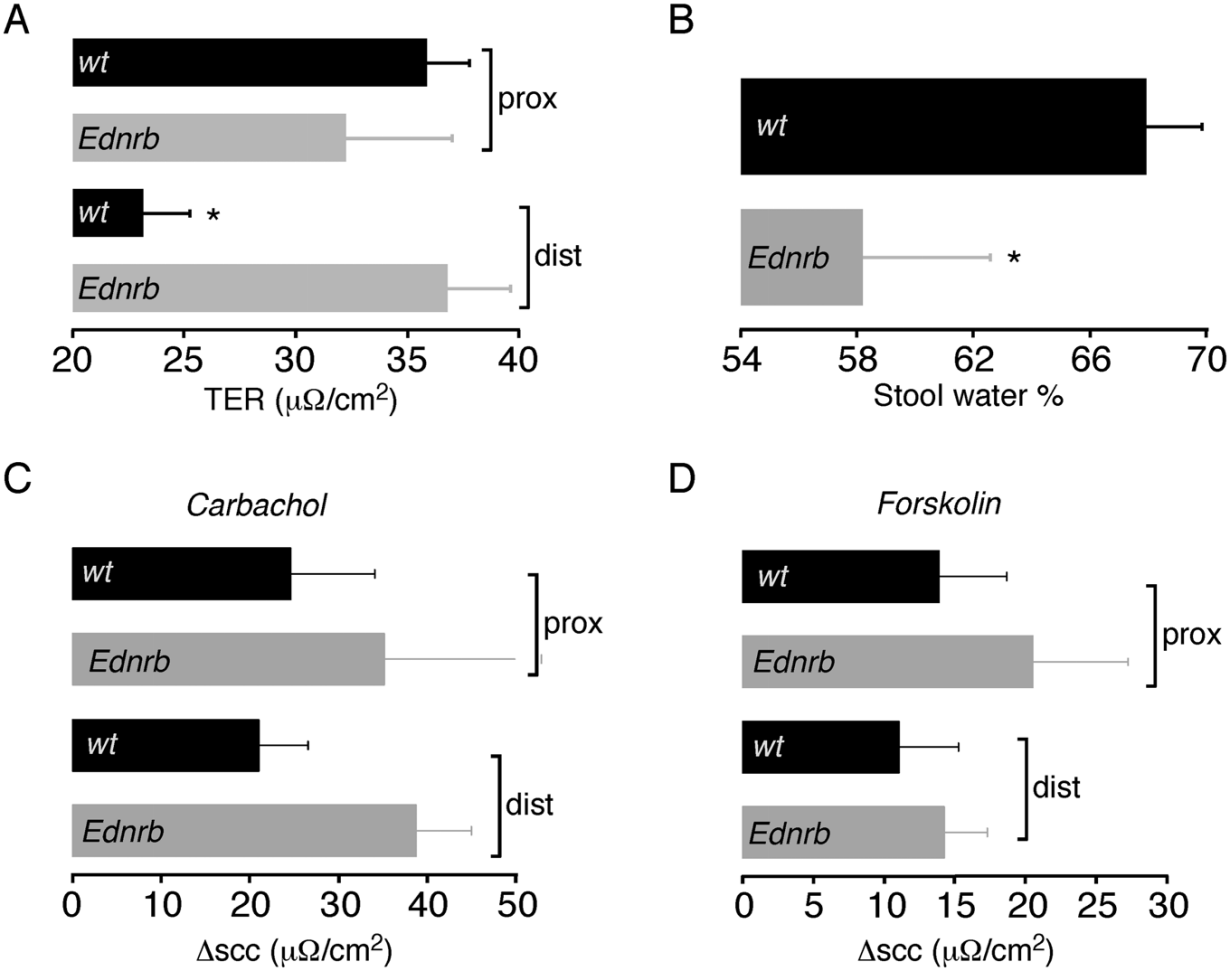
Colon functional properties in Ednrb^−/−^ mice. A. Transepithelial resistance (TER) in distal and proximal colon from WT (n = 8) and Ednrb^−/−^ mice (n = 5). B. Stool water content (%) in distal colon from WT (n = 7) and Ednrb^−/−^ mice (n = 7). C. Change in short-circuit current (scc) after addition of carbachol (100 µM) to proximal (prox) and distal (dist) colon from WT (n = 7) and Ednrb^−/−^ mice (n = 5). D. Change in short-circuit current (scc) after addition of forskolin (50 µM) to colon from WT (n = 7) and Ednrb^−/−^ mice (n = 5). *p<0.05; TER = transepithelial resistance; scc = short-circuit current.

Given the changes seen in the structure of the epithelium, we examined the functional properties of the colonic mucosa. Electrophysiological measurements were performed on whole mount distal and proximal colon from Ednrb^−/−^ and WT mice at P18–24. Mouse models in general show variable degrees in the extent of aganglionosis along the length of the intestinal tract. Previous studies have shown that the distal 3 cm of colon in Ednrb^−/−^ mice is aganglionic and we therefore used distal colonic tissue <3 cm from the rectum (1) and harvested our proximal colon tissue 1–4 cm distal to the cecum to ensure ganglionic colon tissue for comparison. In order to look at overall barrier function in the colon, transepithelial resistance (TER) measurements were performed. Fig. 3A shows that TER was similar in proximal colon of WT and mutant animals. In contrast, the distal mutant colon had a significantly increased TER as compared to WT. More detailed analysis was done to look at the progression of TER over time. After an initial equilibration period following tissue harvesting, colonic TER in WT mice is stable and then steadily declines over time as the tissue integrity is compromised. This decrease in TER was not different between WT and Ednrb^−/−^ distal colon (6.3 vs 5.1 µA/cm^2^).

In addition to barrier function, another colonic property associated with ENS activity is the stimulation of epithelial chloride secretion from crypt enterocytes [27], [28]. In WT colon, elevation of intracellular calcium levels via muscarinic receptors, or increased intracellular cAMP levels via activation of adenylate cyclase, leads to activation of luminal membrane chloride channels and subsequent epithelial chloride secretion. To investigate this, short-circuit current (scc) measurements were performed on Ednrb^−/−^ and WT colon after stimulation with either carbachol (Ca^2+^) or forskolin (cAMP). Fig. 3C and 3D show that carbachol and forskolin stimulated similar increases in short-circuit current in both mutant and WT colon and in both distal and proximal segments. The primary function of the colon is fluid absorption and subsequent fecal dehydration. We measured the water content of stool obtained from the distal colon, and found that Ednrb^−/−^ mice have significantly reduced stool water content compared to WT (Fig. 3B).

### Genes Associated with Goblet Cell Differentiation are Altered in the Distal Colon of Ednrb^−/−^ Mice and HSCR Patients

**Figure 4:**
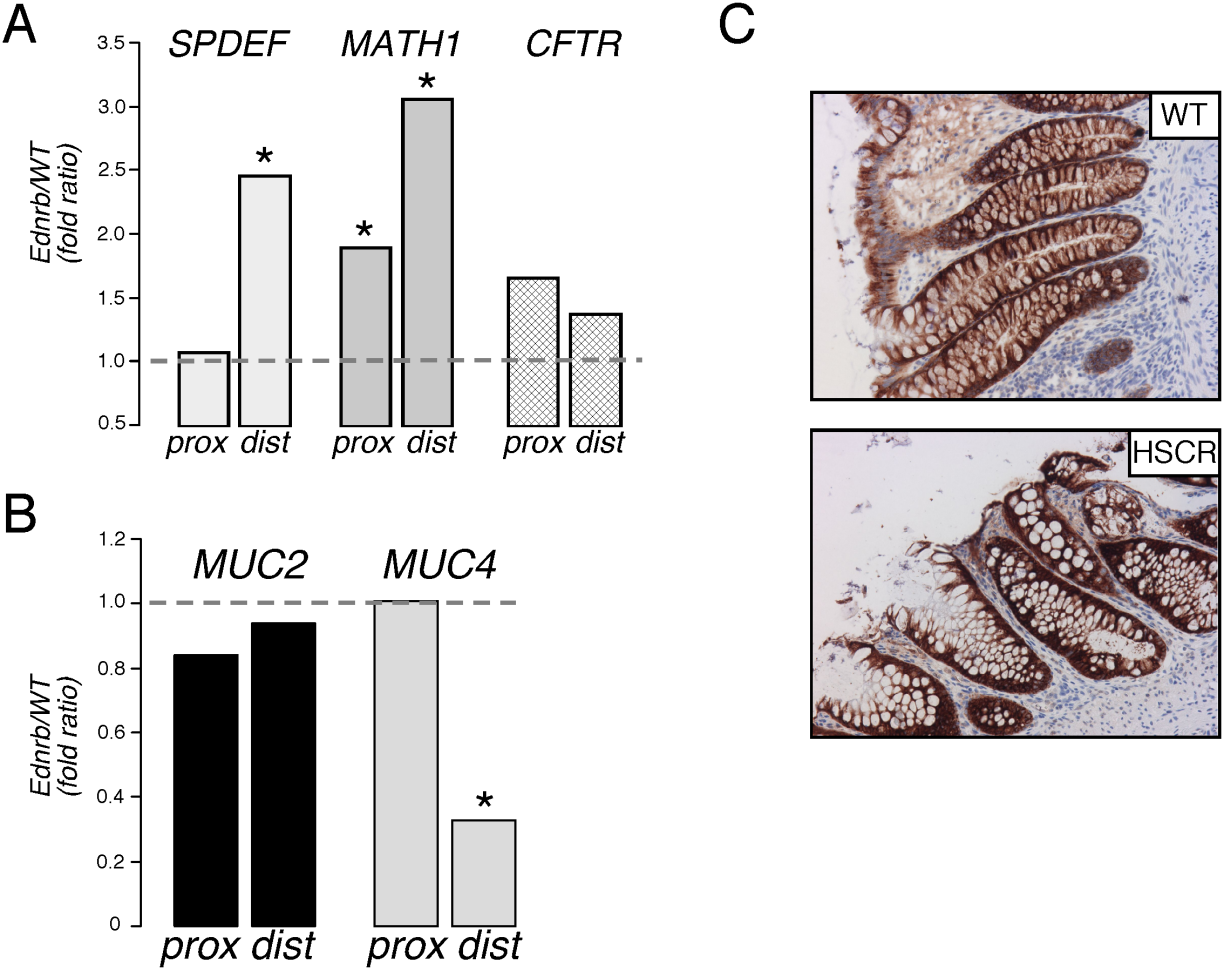
Goblet cell gene expression. A. Muc2 and Muc4 expression by qPCR in mouse proximal (prox) and distal (dist) colon. Data are shown as expression in Ednrb^−/−^ mice relative to WT mice (n = 3). Dotted line (ratio = 1) indicates equivalent expression. B. SPDEF, MATH1, and CFTR transcript expression in mouse colon. C. Representative images show Muc4 staining of crypt cells from control and HSCR colon. *p<0.05.

As goblet cell parameters and overall proliferation appeared to be an early difference in Ednrb^−/−^ colon, the relative expression of factors known to be important in the progression and activation of secretory cell progenitors at the base of colonic crypts and in the differentiation of goblet cells were examined by qPCR. As shown in Fig. 4A, in the distal colon, expression of SPDEF mRNA was 2.6 fold greater in Ednrb^−/−^ vs WT. Similarly Math1 mRNA was 1.9 fold increased in the distal mutant colon. In contrast, although Math1 expression was increased in proximal mutant colon (2.1 fold), SPDEF expression was the same. No difference was observed in transcript levels of the cystic fibrosis transmembrane conductance regulator (CFTR).

Since mucins represent the major protein product of mature goblet cells, the expression of specific mucin subtypes was analysed. The two major categories of mucin proteins are secreted gel-forming mucins (Muc2, Muc5AC), of which Muc2 is the most important in the mouse colon, and the membrane-bound mucins (Muc1, Muc4). Fig. 4B shows that mRNA expression of the secreted mucin Muc2 was similar between Ednrb^−/−^ and WT mice. In contrast, the membrane-bound mucin Muc4 was significantly decreased (3.1-fold) in mutant colon.

Differences in mucin subtype expression in HSCR patient tissue were also studied by immunohistochemical staining of tissue sections. Qualitatively, and mirroring the changes in Ednrb^−/−^ mice, Muc2 staining was similar in colonic crypts between normal and HSCR tissue (not shown), whereas Muc4 staining appeared reduced in HSCR goblet and epithelial cells (Fig. 4C).

### Increased Mucus Viscosity in the Distal Colon of Ednrb^−/−^ Mice

**Figure 5:**
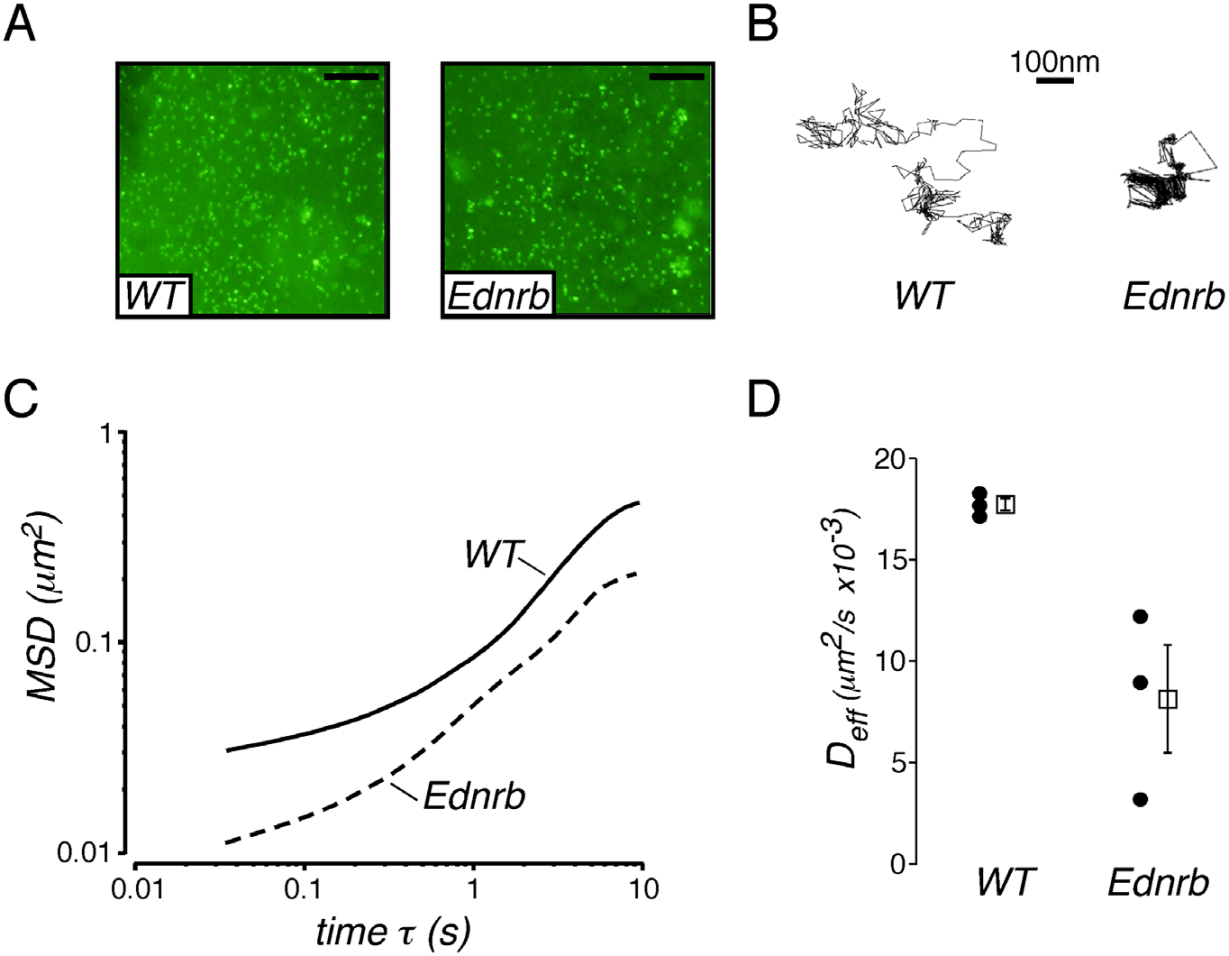
Transport of microspheres in mucus on explanted Ednrb^−/−^ and WT mouse colon. A. Images from WT and Ednrb^−/−^ surface mucus showing equilibration of 200 nm fluorescent particles throughout the mucus layer. B. Representative 20-second trajectories of particles in WT and Ednrb^−/−^ mucus. C. Ensemble averaged mean-squared displacement (MSD) for carboxylate-modified particles in WT and Ednrb^−/−^ mucus as a function of time. D. Ensemble effective diffusion coefficients of particles at a time scale of 10 seconds in WT and Ednrb^−/−^ mice. Black dots represent individual experimental averaged runs, and open squares represent the mean ± SEM. Scale bar = 20 µm (A).

Barrier properties of mucus from Ednrb^−/−^ and WT mice were compared by analyzing passive diffusion of microparticles in intact mucus on colon epithelium using the multiple particle tracking (MPT) technique. In MPT, microscopic motion of hundreds of individual particles is tracked and analyzed to reveal important information pertaining to particle-environment interactions [29]. Fluorescent carboxylate-modified polystyrene microspheres 200 nm in diameter were allowed to diffuse into the colonic mucus. Distribution of microspheres across the mucosal surfaces appeared uniform after approximately 90 min of incubation (Fig. 5A). Analysis of particle transport revealed that particle diffusion was hindered in Ednrb^−/−^ mucus relative to WT, as evidenced by constrained trajectory profiles (Fig. 5B). Particle trajectories were analyzed to calculate time-dependent ensemble mean-squared displacements (MSD) and effective diffusivities (D_eff_) (Fig. 5C, D). The lower MSD and D_eff_ values in Ednrb^−/−^ mice support more hindered transport and enhanced barrier properties in Ednrb^−/−^ relative to WT mucus. Effective diffusivities of microspheres in Ednrb^−/−^ mice were ∼2-fold lower than in WT at a time scale of 10 seconds.

## Discussion

The overall aim of this study was to investigate changes to the colonic epithelium in the setting of congenital aganglionosis that may suggest possible etiologies for HAEC. A number of theories have emerged to explain the pathogenesis of HAEC. As with other inflammatory bowel conditions such as Crohn’s disease and ulcerative colitis, these theories have primarily focused on defects in colonic barrier function, innate immunity and the microbial milieu. The occurrence of enterocolitis both in HSCR patients and in mouse models of colonic aganglionosis suggest that there are fundamental changes to colonic structure and function that occur either in conjunction with defective neural crest migration or that result from the absence of enteric neuronal or glial function in the mucosa and submucosa. We obtained biopsy tissue from HSCR patients with minimal or no inflammation to see if there were obvious structural changes associated with aganglionosis. A common feature of the human tissues was increased numbers of goblet cells. Although the mean ages of the HSCR and control patients were similar, given the range of ages and clinical conditions in the patient biopsies we wanted to investigate possible epithelial differences without confounding by developmental or condition-specific changes. We therefore undertook a detailed analysis of the colon from neonatal Ednrb^−/−^ mice prior to the onset of inflammation or any phenotypic signs of enterocolitis. Ednrb^−/−^ mice exhibit many of the features found in patients with HSCR, including defective colonic motility and development of enterocolitis [30].

The primary function of the mammalian distal colon is the salvage of water and electrolytes from the luminal intestinal contents and the dehydration and storage of feces. We found increased fecal dehydration (reduced stool water content) in Ednrb^−/−^ distal colon, suggesting that fluid absorptive function remained intact. It seems likely that the reduction in stool water content is due to increased resident time in the colon secondary to impaired motility in mutant mice, rather than increased fluid absorption per se. However, a previous report found increased sodium and chloride transport across the rectum of HSCR patients, suggesting that there may be enhanced salvage of fluid and electrolytes in HCSR [31]. We also found that the epithelial chloride secretory response to both carbachol and forskolin remained intact in Ednrb^−/−^ distal colon. The carbachol response indicates that the epithelium retains the appropriate muscarinic receptors (M2) required for cholinergic chloride secretion. Although there is no reported data on epithelial muscarinic receptors in HSCR, studies have shown reduced intestinal muscle expression of certain subtypes of muscarinic receptors in keeping with the finding of hypertrophied acetylcholinesterase positive fibers in HSCR [32]. Our results in the Ednrb^−/−^ mouse colon agree with a previous study that showed an intact colonic carbachol mediated chloride secretion in HCSR patients [33]. This suggests that the Ednrb^−/−^ mouse model appears to be a useful model to study the details of colonic epithelial absorptive and secretory function in the absence of the ENS.

Changes in barrier function properties have been proposed as a possible intrinsic defect in HSCR colon. Barrier function is comprised of the luminal mucus layer as well as the epithelium itself. Transepithelial electrical resistance (TER) has been widely used as a proxy for epithelial barrier function and there are no published data on progression of TER in either mouse models or human HSCR. Surpisingly, TER was higher in the aganglionic mouse colon, suggesting that electrical integrity, which is largely a function of epithelial tight junction properties, was not only intact in the presence of aganglionosis, but was even enhanced. A number of possibilities could explain this result, including changes in tight junction protein composition, faster maturation of epithelial junctions in the neonatal mutant mouse and others. A useful next step will be a detailed study of the progression of epithelial resistance, including macromolecular paracellular permeability, in the Ednrb^−/−^ colon.

The main structural difference found in our study in both HSCR patient tissues and Ednrb^−/−^ distal colon was increased goblet cell numbers. Additionally, goblet cell maturation appeared to be altered as suggested by the loss of the membrane bound mucin Muc4. These changes were accompanied by increases in Math1 and SPDEF, important intestinal epithelial secretory cell differentiation factors, suggesting that aganglionosis is associated with changes to the normal developmental program that drives the generation and constant renewal of the colonic crypt epithelium. Goblet cell parameters were conversely lower in the proximal colon, with increased Math1 but similar SPDEF expression, although it is unclear how the balance of proliferation versus differentiation of goblet and epithelial cells relates to HAEC susceptibility. While we did not find Muc2 levels altered in the epithelium, in contrast to a previous study [34], it may be that functionally secreted levels of Muc2 may be altered, which would require more detailed analysis of colonic secretions in the mutant mice as well as in fecal samples from patients. Qualitatively we did observe reduced amounts of both neutral and acidic mucins within HSCR crypt goblet cells. This may reflect altered maturation of HCSR goblet cells or, as suggested by a previous study, increased secretion of these mucins into the crypt lumen [35].

The mucus layer overlying the colonic epithelium has multiple functional roles, including providing an initial barrier to pathogens, altering selection pressures on commensal microbiota and lubrication of intestinal contents. The layer itself resembles a mesh of mucin glycoproteins, and anti-bacterial and signaling proteins within an aqueous environment. The alterations in goblet cell number and function found in mutant mice appear to produce a significantly different mucus layer environment, as shown by the altered diffusivity of 200 nm particles found in the particle tracking studies. These changes may be due to altered macro-scale visco-elastic properties and/or nano-scale changes in mucin interactions resulting in a reduced effective mesh pore size. Alternatively, the changes may reflect altered interaction between mucus proteins and the carboxylate surface groups of the particles. Our previous studies have shown significant alterations in the commensal milieu in aganglionic colon [26], which we speculate may be related to the altered epithelial and goblet cell proliferation and differentiation and subsequent altered mucus environment found in this study. Altered commensal signaling is well-known to increase colonic susceptibility to injury [36] and this defective regenerative response has been linked to dysregulated proliferation and differentiation of epithelial cells in a number of models [37], [38], [39], [40]. Recent in vitro evidence also supports our data that the ENS has an important role in controlling epithelial cell proliferation [41].

In summary, these results suggest that aganglionosis is associated with changes to goblet cell differentiation and functional expression of mucin sub-types prior to significant inflammation in the distal colonic epithelium. Additionally, these changes result in a functionally altered mucus layer with significantly hindered movement of nanoparticles. Despite these changes, distal colonic function in terms of fluid absorption, secretion and epithelial barrier function, are largely preserved in Ednrb^−/−^ mice. Further studies will aim to elucidate the mechanisms by which defective goblet cell function in conjunction with an altered colonic microbiome and/or innate immune signaling pathways result in HAEC. Restoration of normal goblet cell function or mucus layer properties in both innervated and non-innervated colonic epithelium may represent a therapeutic strategy for prevention of HAEC.

## Materials and Methods

### Human Colon Tissue

All human tissue samples were obtained from the archives of the Department of Pathology at Massachusetts General Hospital with approval from the Massachusetts General Hospital Institutional Review Board (Partners Human Research committee). Protocol number: 2003-P-000460/20. The Institutional Review Board waived written donor consent. Colonic samples from patients with Hirschsprung’s disease (n = 13) were from the resected aganglionic segment at the time of their pull-through operation. Samples from age-matched controls (n = 4) were from rectal biopsies obtained during a work-up for constipation to rule-out Hirschsprung’s disease. The ages and extent of aganglionosis of the patients are summarized in Table 1.

Sections of 6 µm thickness were prepared from formalin-fixed, paraffin-embedded tissues. H&E, alcian blue, PAS, and Muc4 stains on human tissue sections were done using standard histology protocols using automated sectioning and staining equipment (Leica Stainer XL for H&E and Ventana Bench Mark Special stainer for alcian blue and PAS) Anti-Muc4 antibody was from Santa Cruz Biotech(Dallas).

### Mouse Tissue

Experiments were approved by the Massachusetts General Hospital Institutional Animal Care and Use Committee (MGH IACUC OLAW Assurance number: A3596-01, Protocol number: 2009N000239). Ednrb^tm1Ywa^ mice on a hybrid C57BL/6J-129Sv background (JAX#003295) were maintained on a 12-h light–dark cycle at 25°C and had unlimited access to water and standard rodent chow (Prolab Isopro RMH 3000 Irradiated; PMI Nutrition International, St. Louis, MO, USA). Homozygous mutant mice are identified by their white coat color and the genotype confirmed by polymerase chain reaction. Mice were weaned at postnatal day 21 (P21). Controls consisted of colon tissue from homozygous WT animals. Mice were euthanized by CO2 asphyxiation.

The colon was removed and fixed with 4% paraformaldehyde. Cryosections (10 µm thick) were collected on Probe-on poly-L-lysine-coated slides (Fisher Scientific, Pittsburgh, PA). For analysis of cell division, immunohistochemistry was performed using primary antibody against PCNA (NeoMarkers, Fremont, CA, 1∶100 dilution), biotinylated goat anti-mouse IgG secondary antibody (Vector Labs, Burlingame, CA, 1∶200 dilution), avidin-biotinylated peroxidise complex (Vectastain Elite ABC kit, Vector Labs), and the Perodixase Substrate Kit (DAB, Vector Labs). Slides were examined under a Nikon 80i microscope and photographed with a Spot camera.

For goblet cell labelling, sections were incubated in 3% acetic acid (Fisher Scientific), followed by Alcian blue (Sigma-Aldrich, St. Louis, MO) for 30 minutes at room temperature. After rinsing, Nuclear Fast Red (Vector Labs) was added for 5 minutes at room temperature, and then the slides were rinsed in water followed by 95% and 100% alcohol.

### Histological Analysis

Ednrb^−/−^ mice: For PCNA staining, individual crypts were analysed from images (20x) obtained from stained sections. At least 3 crypts per image and 3 images per section were examined by a blinded assessor from n = 5 mice per genotype. Goblet cell parameters were analyzed using a square region of interest (ROI) of arbitary size (512×512 pixels). Numbers of goblet cells within the ROI and size of the ten largest goblet cells were recorded.

HCSR patient samples: Goblet cells per crypt were counted and normalized to total crypt length. At least 3 crypts per image and 3 images per section were examined from patient samples. Size of goblet cells was measured from all counted goblet cells and averaged. Analysis was done blinded to section origin and patient information. All images were analyzed using Image J image analysis software (NIH, Wayne Rasband).

### Electrophysiological Measurements

Mouse colon was harvested after CO_2_ asphyxiation and cervical dislocation. The colon was washed with ice-cold Krebs buffer, opened along the mesenteric border. Distal colonic tissue (<3 cm from the rectum) and proximal colon tissue (1–4 cm distal to the cecum) was mounted in a micro-Ussing chambers (area 0.2 cm^2^; Physiological Instruments, San Diego, CA). Hemichambers were filled with oxygenated Krebs-bicarbonate solution and tissues were incubated for 30 mins prior to recording measurement. Short-circuit current was recorded using a DVC-1000 voltage-clamp (World Precision Instruments) with Ag/AgCl electrodes and 1 mol/L KCl agar bridges. Agonists/inhibitors (0.1% DMSO final) were added to hemichambers. Transepithelial resistance was calculated from measured current change after 5 mV voltage pulses.

### Fecal Water Content

Stools were collected from the distal colon of P17–P22 WT (n = 7) and mutant (n = 7) mice at the time of sacrifice. Stools were weighed then dehydrated overnight at 55°C and weighed again. The percentage of water content  =  [(wet weight-dry weight)/wet weight]*100.

### Mouse Colon Quantitative PCR (qPCR)

Total RNA was extracted using TRIzol Reagent (Life Technologies, Grand Island, NY) and cDNA generated with ThermoScript (Invitrogen). The specificity of amplification products was verified by electrophoresis on an agarose gel. qPCR was performed on a CFX96 Real-Time PCR Detection System. The expression level of each gene was normalized to *Gapdh*. Samples were analyzed in triplicate from 3 different mouse colon specimens.

The following oligonucleotide primer pairs were used:

*Muc2*: (F) CAAGTGATTGTGTTTCAGGCTC; (R) TGGAGATGTTCTTGGTGCAG
*Muc4*: (F) GACAAAGCACCAATTCCATCC; (R) CCTTAGAGTTGCTGGTGATCTC
*Spdef*: (F) ACGTTGGATGAGCACTCG; (R) CCATAAAAGCCACTTCTGCAC
*Math1*: (F) GCTTATCCCCTTCGTTGAACTG; (R) CTCTTTTACCTCAGCCCACTC
*Cftr*: (F) CCGATCAGTTCTCAGTAAGGC; (R) ATCGCTTCTATCCTGTGTTCAC
*Gapdh*: (F) CTTTGTCAAGCTCATTTCCTGG; (R) TCTTGCTCAGTGTCCTTGC

### Single Particle Tracking

#### Preparation of nanoparticles

Particle suspensions were prepared using 200 nm carboxylate-modified yellow-green fluorescent microspheres (FluoSpheres, Invitrogen Molecular Probes, Carlsbad, CA). Microsphere solutions (2% solids in distilled water with 2 mM azide) were diluted in maleate buffer consisting of 100 mM Trizma, 10 mM CaCl_2_, 65 mM NaCl, 40 mM NaOH and 3 mM NaN_3_ mimicking gastrointestinal tract contents to yield a final particle concentration of 0.0025% wt/vol.

#### Collection and preparation of intestinal tissue

Intestinal tissue segments were collected from WT and Ednrb^−/−^ type mice ranging in age from P11–P20. The most proximal and most distal 1 cm of colon were removed and placed in a chamber on a glass slide formed by attached silicone isolators (1.6 mm depth and 13 mm diameter, Grace Bio-Labs). Colon sections were cut open such that the mucosal surface was exposed, and 5 µl of diluted particle solutions carefully pipetted onto the mucus surface. Samples were covered in a humid chamber, protected from light, and left at room temperature for 90 min before microscopy.

#### Real-time multiple particle tracking

Particles were tracked using an Olympus DP70 digital color camera (Olympus, Center Valley, PA) mounted on an inverted Olympus IX51 microscope with attached X-Cite 120 fluorescence illumination system (EXFO, Mississauga, Ontario, Canada). 20 second particle diffusion videos were captured using Olympus DP imaging software, with n≥100 particles analyzed in each excised section (proximal or distal) of the colon. Particle trajectories were generated using tracking algorithm of the ParticleTracker ImageJ plugin [42]. Mean-squared displacements (MSD) and effective diffusivities (D_eff_) were determined as previously described [43], [44].

### Statistics

Experimental groups were analyzed by unpaired t-test for single comparisons and ANOVA with post-hoc Tukey-Kramer analysis for multiple comparisons. p-values are indicated in the text and figure legends.
